# Chest radiographs of cardiac devices (Part 1): Lines, tubes, non-cardiac medical devices and materials

**DOI:** 10.4102/sajr.v23i1.1729

**Published:** 2019-07-29

**Authors:** Rishi P. Mathew, Timothy Alexander, Vimal Patel, Gavin Low

**Affiliations:** 1 Department of Radiology and Diagnostic Imaging, Faculty of Medicine and Dentistry, University of Alberta, Edmonton, Canada

**Keywords:** Chest radiographs, endotracheal tube, tracheostomy tube, nasogastric tube, central venous catheter, Swan Ganz catheter, intercostal drainage tube

## Abstract

Chest radiographs (CXRs) are the most common imaging investigations undertaken because of their value in evaluating the cardiorespiratory system. They play a vital role in intensive care units for evaluating the critically ill. It is therefore very common for the radiologist to encounter tubes, lines, medical devices and materials on a daily basis. It is important for the interpreting radiologist not only to identify these iatrogenic objects, but also to look for their accurate placement as well as for any complications related to their placement, which may be seen either on the immediate post-procedural CXR or on a follow-up CXR. In this article, we discussed and illustrated the routinely encountered tubes and lines that one may see on a CXR as well as some of their complications. In addition, we also provide a brief overview of other important non-cardiac medical devices and materials that may be seen on CXRs.

## Introduction

Among the various imaging modalities available for assessing the cardiothoracic system, the chest radiograph (CXR) is the most commonly used. In addition, the CXR is very useful in assessing the numerous tubes, lines, medical devices and materials, as well as for identifying any equipment-related complications. The American College of Radiology (ACR) recommends a CXR immediately after the placement of a medical tube, catheter or device, to check for malposition or for intra- or post-procedural-related complications. As any medical device has the potential for coiling, mispositioning, kinking or fracturing and malfunctioning, the complications that may ensue are often not immediately apparent clinically. Hence, it is of utmost importance to identify these on the immediate post-procedural CXR or follow-up CXRs for the physician or surgeon to replace or reposition these devices.^1^ The objective of this article is to provide a comprehensive review of the numerous tubes, lines and non-cardiac medical devices or materials that may be seen on CXRs, their appearance on CXRs and how to evaluate for their accurate placement, as well as to be aware of associated complications that need to be considered.

## Tracheal and oesophageal tubes and lines

### Endotracheal tube

**FIGURE 1:**
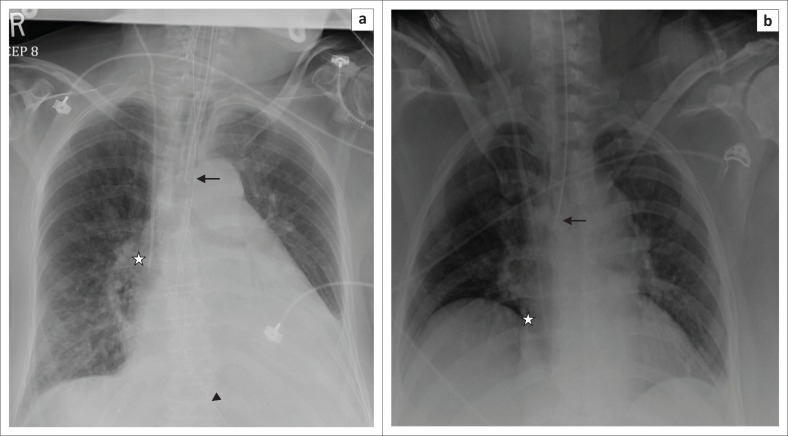
(a) Magnified chest radiograph showing an accurately placed endotracheal tube with its distal end (arrow) located above the carina. Other lines and tubes visualised are the right internal jugular vein central venous catheter (star) and the nasogastric tube (arrow head); (b) chest radiograph showing a malpositioned endotracheal tube with its tip in the right main bronchus (arrow). A central venous catheter is noted in the right atrium (star).

The endotracheal (ET) tube is inserted for maintaining the patency of the airways or to provide airway support. The accurate positioning of the ET tube is assessed by calculating the distance of the tip of the ET tube from the carina. An ideal position would be 5 cm above the carina (Figure 1a) with the patient’s head in a neutral position, taking into consideration that neck extension or flexion would lead to 2 cm of movement upwards or downwards. In cases where the carina is not visible on the CXR, the aortic ‘knob’ can be used as a useful landmark. The carina is just distal to the aortic arch, and if the ET tube is just above the aortic arch, it can be considered to be in a safe position midway between the vocal cords and the carina.^2^ If the carina is not visible an additional landmark that could be utilised is by confirming that the ET tip lies between the T2 and T4 vertebrae.^3^

A malpositioned ET tube (Figure 1b) is the most common complication associated with ET tube insertion. Complications associated with a wrongly placed cephalad ET tube include inefficient ventilation and distension of the stomach and vocal cord injury caused by the inflated cuff. Hence, the ET tube tip should be at least 3 cm caudal to the vocal cords. If the ET tube is advanced too caudally, the tube can selectively intubate one of the main bronchi, usually the right, leading to segmental or complete collapse of the lung. Accidental intubation of the oesophagus is a potentially fatal complication. In such cases, a frontal CXR may show the ET tube lateral to the tracheal air column and the identification of a column of air lateral to the trachea along with an overdistended stomach.^4^

### Tracheostomy tube

**FIGURE 2:**
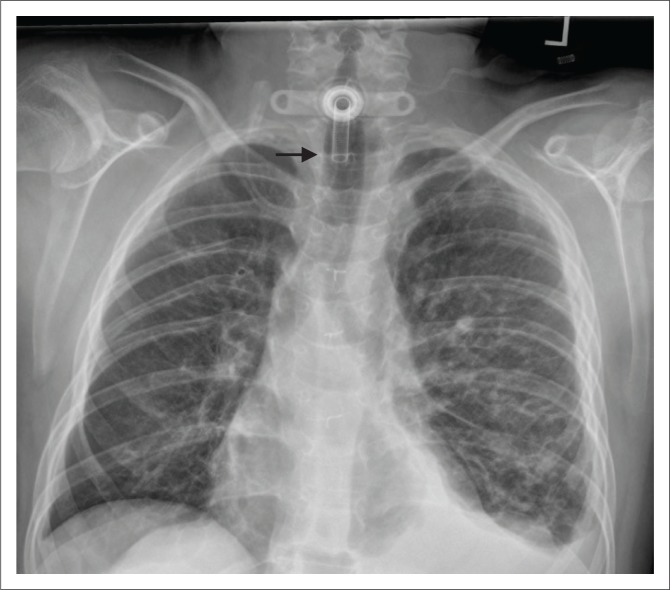
Chest radiograph showing an accurately positioned tracheostomy tube (arrow).

Tracheostomy tubes (Figure 2) are inserted for long-term ventilation. Its distal end should be midway between the stoma and the carina at the level of third thoracic vertebra. When compared with the ET tube, the position of the tracheostomy tube is maintained with neck flexion and extension.

The width of the tube should not exceed two-thirds of the tracheal width, and the cuff should not distend the tracheal wall. Complications associated with tracheostomy tubes include subcutaneous emphysema, haematoma and pneumomediastinum. Granulation tissue formation and fibrosis at the site of the stoma can lead to tracheal stenosis.^1,4^

### Nasogastric tube

**FIGURE 3:**
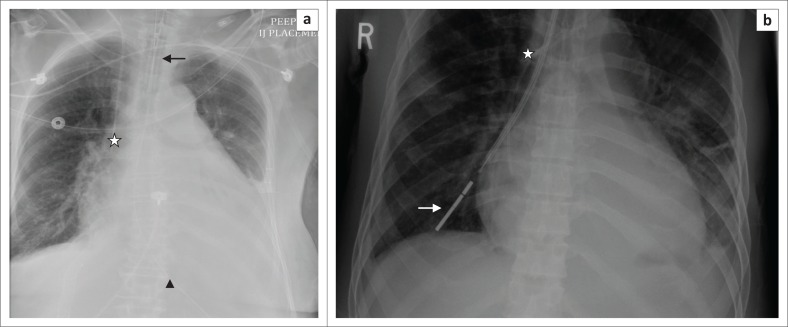
(a) Chest radiograph showing an accurately positioned nasogastric tube with its proximal side hole (arrowhead) and tip located beyond the gastroesophageal junction. In addition, an endotracheal tube (black arrow) and a right internal jugular vein central venous catheter (star) are noted on this chest radiograph; (b) a malpositioned Dobhoff tube (white arrow) in the right main bronchus. Additionally, a left central venous catheter (star) is noted with its tip in the superior vena cava.

Nasogastric (NG) tubes are frequently used for suction of stomach contents, administration of medication and for feeding. The NG tube has multiple side holes and a thin radiopaque marker line along one side, enabling it to be identified on a radiographic study (Figure 3a). The proximal side hole is indicated by a short break in this line. Both the upper side hole and tip should be located clearly below the gastroesophageal (GE) junction. An ideal position of the NG tube would be with its tip in the stomach caudal to gastric cardia or at least 10 cm distal to the GE junction.^1,4^ A malpositioned NG tube is the commonest complication and can lead to lung perforation, oesophageal perforation, pneumonia and pneumothorax.^5,6^

*Dobhoff tubes* are flexible tubes with a narrow bore measuring 4 mm in diameter for delivering enteral nutrition. Unlike NG tubes, suction cannot be applied to these tubes, making it useful only for feeding and delivery of drugs. These tubes provide two advantages over the NG tube^1^ because of their narrow bore; they are better tolerated by patients^2^, and the tubes enable post-pyloric feeding. These tubes are inserted into the stomach or duodenum with the aid of a guidewire through the nasal passage, which is then removed once the distal end of the tube is in an acceptable position. The distal end of the tube has a metallic end visible on a CXR. The gold standard for confirming an accurately inserted Dobhoff tube is by radiographic examination. Complications associated with these tubes include accidental insertion into the airway (Figure 3b), and if advanced further into the lungs may lead to trachea-pulmonary complications such as pneumothorax.^7^

**FIGURE 4:**
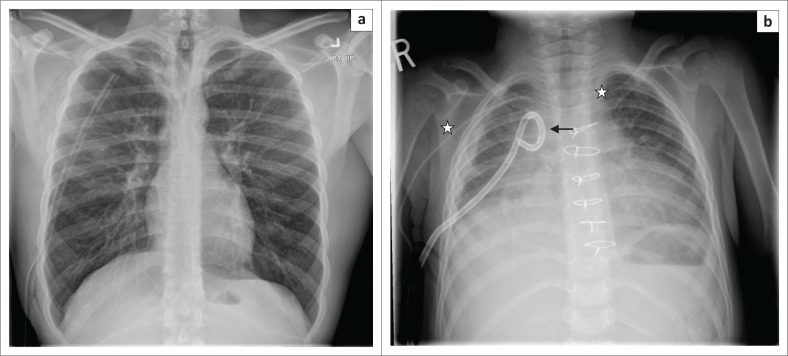
(a) Chest radiograph showing an intercostal drainage (ICD) tube introduced for pneumothorax; (b) a pigtail catheter (arrow) in the right thorax inserted for pleural effusion. Additionally, a malpositioned right peripherally inserted central venous catheter (PICC) [star] is noted with its tip in the left brachiocephalic vein.

**FIGURE 5:**
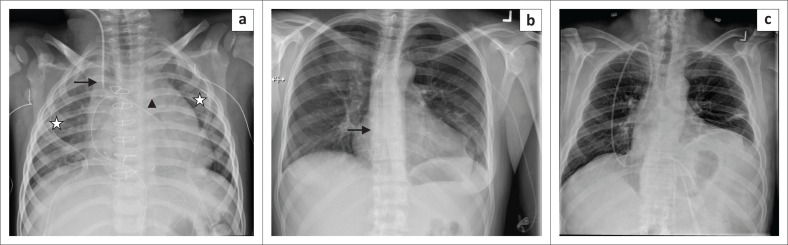
(a) Chest radiograph showing a right central venous catheter (arrow) with its end located in the SVC. In addition, bilateral intercostal drainage tubes (stars) and mediastinal drainage tube (arrow head) are noted; (b) chest radiograph showing a left-sided, peripherally inserted central catheter with its tip (arrow) in the right atrium; (c) a tunnelled right central venous catheter.

**FIGURE 6:**
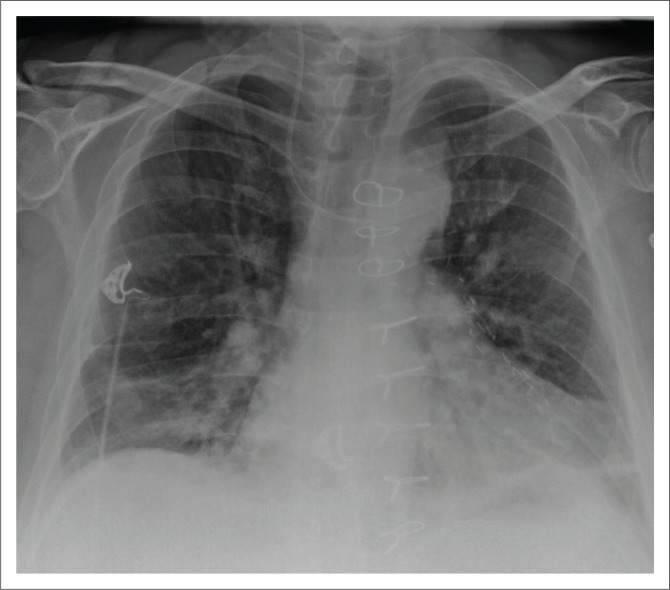
Magnified chest radiograph showing a malpositioned right central venous catheter in the left brachiocephalic vein.

**FIGURE 7:**
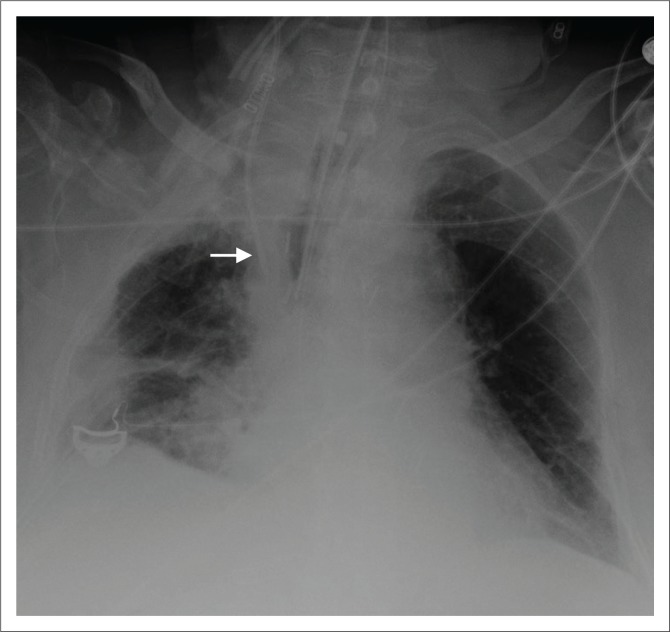
Magnified chest radiograph showing a widened superior mediastinum secondary to central venous catheter induced haematoma.

**FIGURE 8:**
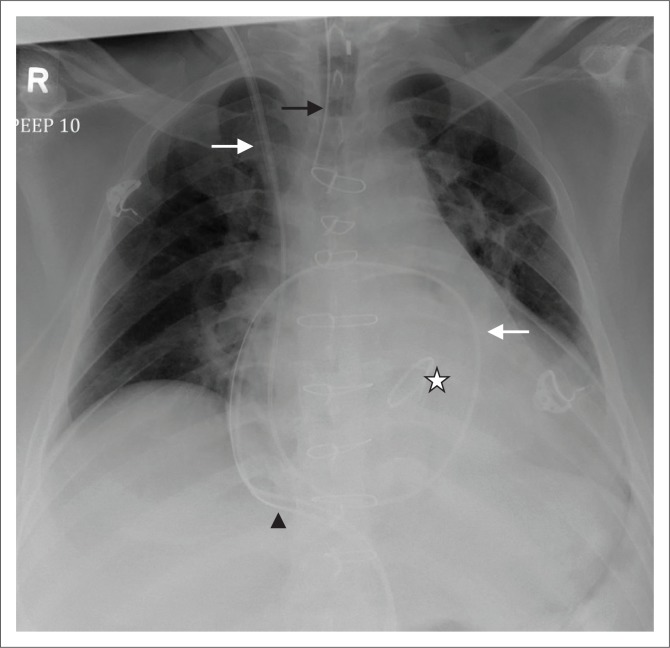
Chest radiograph showing a Swan Ganz or pulmonary artery catheter (white arrow), with its tip in the right pulmonary artery. The other devices on this chest radiograph are an endotracheal tube (black arrow), a mediastinal drainage tube (arrow head) and a prosthetic aortic valve (star).

### Chest drainage or inter costal drainage tubes

An intercostal drainage tube, also called tube thoracostomy, is commonly inserted for pneumothorax, haemothorax, pleural effusion, and empyema. It also enables instilling antibiotics (after port pneumonectomy for empyema), fibrinolytics, saline and sclerosing agents (pleurodesis).

**FIGURE 9:**
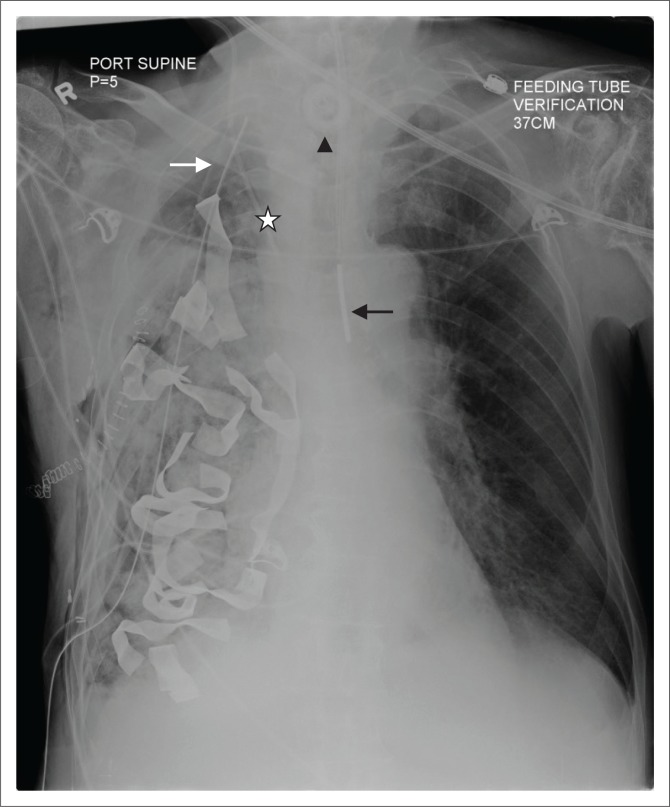
Haemostatic packing in the right thorax in a 67-year-old male who underwent pneumonectomy. In addition, visualised are a tracheostomy tube (arrow head), a malpositioned Dobhoff tube (black arrow) in the mid-oesophagus, a right central venous catheter with its tip in the mid-SVC (star) and an intercostal drainage tube (white arrow).

Both anteroposterior (AP) and lateral CXRs are required for optimal assessment of an intercostal drain (ICD) tube. Like the NG tube, an ICD tube also contains side holes that can be identified by the interruption of its radiopaque outline on a CXR. The side holes of the ICD tubes should be in the thoracic cavity and not in the chest wall or outside the chest. Some of the complications associated with ICD tubes that may be seen on CXRs include: malpositioning (commonest complication), tube kinking, subcutaneous emphysema, pneumothorax and retained catheter fragment.^8^ A properly placed tube for pneumothorax (Figure 4a) should orient antero-superiorly, and an adequately positioned tube for pleural fluid evacuation should be directed posterior-inferiorly. The numerous locations where the tube may be malpositioned include the interlobar fissure, lung parenchyma and subcutaneous soft tissues.^9^ There are several tubes available for thoracostomy and these include the traditional wide bore (>28 F) catheters and the newer popular pigtail catheters (10–14F) (Figure 4b) inserted by guide wires.^10^

**FIGURE 10:**
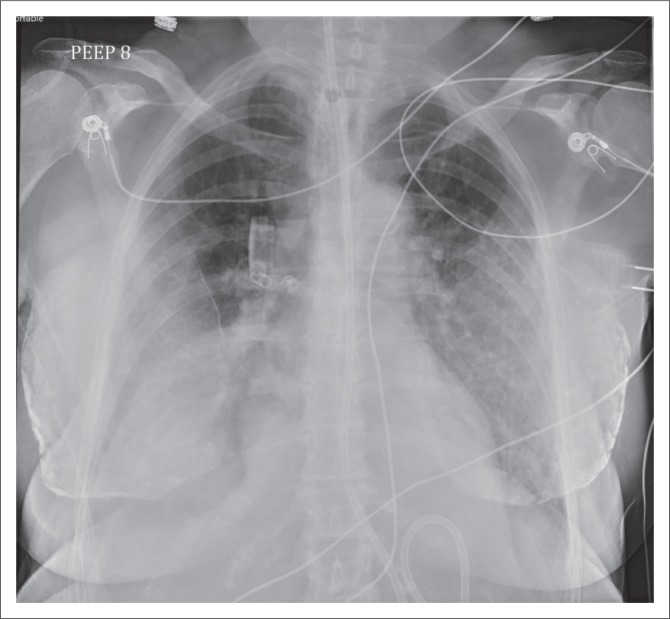
Chest radiograph showing heavily calcified breast implants obscuring the lung bases.

**FIGURE 11:**
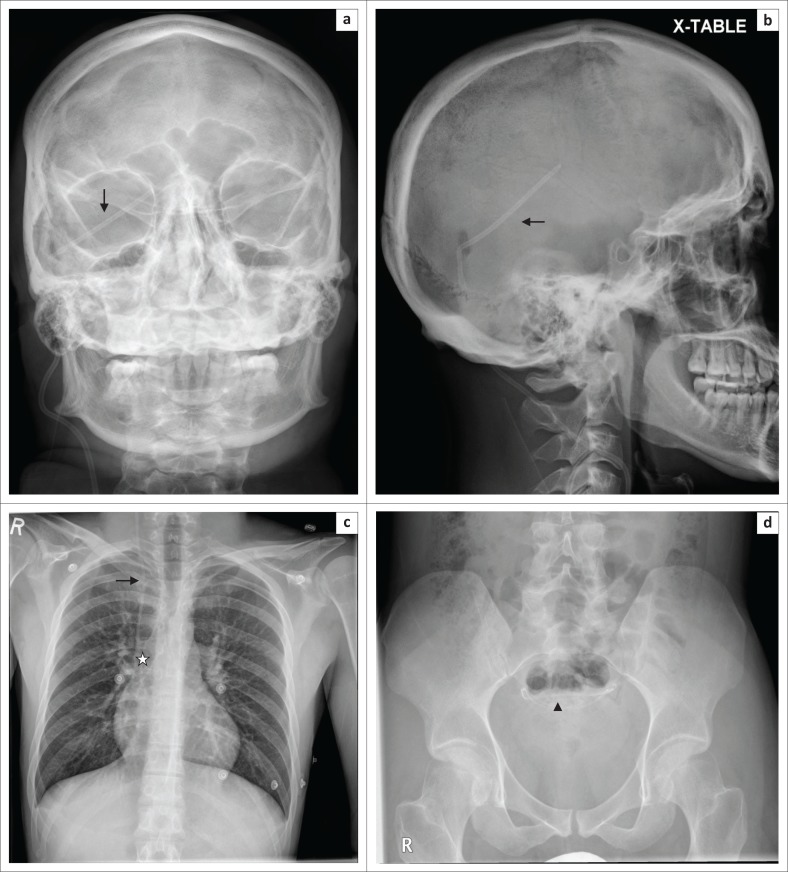
(a) Frontal radiograph of the head and neck of a typical shunt series showing the proximal catheter (arrow) in the right lateral ventricle; (b) lateral radiograph of the head and neck of a typical shunt series showing the proximal catheter (arrow) in the lateral ventricle; (c) chest radiograph of a typical (ventriculoatrial) shunt series showing the distal catheter (arrow) terminating at the cavoatrial junction (star) and (d) magnified image of an abdominal radiograph showing an abandoned ventriculoperitoneal shunt catheter in the pelvis (arrow).

### Cardio-vascular lines on chest radiograph

#### Central venous catheter

A central venous catheter (CVC), also known as a central venous pressure (CVP) catheter or a central catheter or line, is a catheter (Figure 5a) that provides access for administering drugs or fluids, monitoring central venous or pulmonary artery pressure, monitoring central venous blood oxygen saturation, transvenous cardiac pacing, access for extracorporeal blood products and interventions. These catheters are usually inserted via the subclavian or internal jugular vein and less frequently via the femoral vein. A peripherally inserted central catheter (PICC) is a CVC of smaller calibre (Figure 5b) that is usually inserted via the antecubital vein and remains in place for months.^2,11,12^ Tunnelled CVCs (Figure 5c) are cuffed catheters that provide long-term intravenous access for a variety of purposes, which include parenteral nutrition, chemotherapy and haemodialysis. The commonest application for this CVC is for haemodialysis in patients with renal failure.^13^

Most clinicians prefer the CVC tip to be positioned at or just above the cavoatrial junction. As the cavoatrial junction may be difficult to identify on a CXR, the most commonly used and reproducible landmark is two vertebral body levels below the carina. An alternative landmark would be the reflection of the SVC contour with the right heart border and the point where the bronchus intermedius intersects the right heartborder.^14^ A lower placement is preferred for cannulations introduced from the left side. The various types of CVCs, potential contraindications and complications are shown in Table 1^12^ and Figures 6 and 7.

**TABLE 1:** A summary of the various central venous catheters, potential contraindications and complications.^12^

Type of line	Site of insertion	Duration	Use	Potential contraindications	Complications
Non-tunnelled	Internal jugular vein, subclavian vein, axillary vein, femoral vein	Short term (several days to 3 weeks)	Difficult intravenous access; infusion of irritant drugs, vasopressors, inotropes; short-term total parenteral nutrition	Coagulopathy	*Mechanical complications*: arterial puncture, intra-arterial placement of the catheter, haemorrhage, pneumothorax, haemothorax, arrhythmia, cardiac tamponade, etc.
Peripherally inserted	Basilic vein, cephalic vein, brachial vein	Medium term (weeks to months)	Difficult intravenous access; blood sampling; medium-term drug administration (for example, antibiotics); administration of irritant drugs (such as chemotherapy); total parenteral nutrition	Thrombocytopenia	*Thromboembolism*: catheter-related thrombus, pulmonary embolism, air embolism
Tunnelled (for example, Hickmann, Groshong)	Internal jugular vein, subclavian vein	Long term (months to years)	Long-term administration of irritant drugs (such as chemotherapy)	Ipsilateral haemothorax or pneumothorax	*Infection*: catheter colonisation or blood stream infection
Totally implantable (such as implanted port)	Internal jugular vein, subclavian vein	Long term (months to years)	Long-term intermittent access (for example, regular hospital admissions with poor intravenous access); administration of irritant drugs (such as chemotherapy)	Vessel thrombosis, stenosis or disruption	*Catheter-related complication*: catheter tip migration and line fracture or embolism.
-	-	-	-	Infection overlying insertion site	-
-	-	-	-	Ipsilateral indwelling central vascular devices	-

*Source*: Smith RN, Nolan JP. Central venous catheters. BMJ. 2013;347:f6570. https://doi.org/10.1136/bmj.f6570

#### Pulmonary artery catheter or Swan Ganz catheter

Pulmonary artery catheterisation involves inserting a catheter (Swan Ganz) through a central vein (mostly the subclavian or jugular vein and rarely through a femoral vein) into the right heart and finally into the pulmonary artery (Figure 8). Its function is to measure cardiac output, stroke volume, mixed venous oxygen saturation, intracardiac pressure and pulmonary wedge capillary pressure. An ideal location of the pulmonary artery catheter (PAC) tip would be in either the right or left pulmonary artery, not extending beyond the proximal interlobar artery, i.e. within 2 cm of the hilum. If the PAC tip extends beyond the large pulmonary arteries complications such pulmonary infarction or injury to small vessels, that is, pseudoaneurysm, can occur. A malpositioned PAC tip within the right ventricle increases the risk for ventricular arrhythmias and cardiac perforation.^11,15,16^ Other complications related to PAC insertion that may be seen on chest radiographs include pneumothorax, haematoma and catheter migration.^16^

**FIGURE 12:**
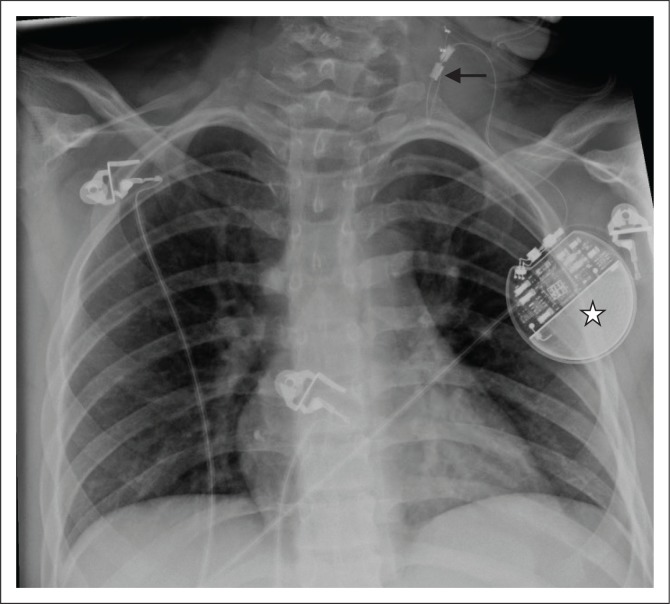
A vagal nerve stimulator, its electrode (arrow) and pulse generator (star) in a 10-year-old male patient diagnosed with Lennox-Gastaut syndrome and suffering from refractory seizures.

### Non-cardiac iatrogenic medical devices and materials

Medical devices and materials such as electrocardiogram (ECG) leads, ventilator tubing, syringes, clamps, temperature sensors, post-coronary artery bypass grafting (CABG) sternotomy wires, surgical clips, orthopaedic implants, gown snaps, etc. are commonly seen on CXRs. Because of its limited scope, we have not included cardiac devices in this article. However, there are other non-cardiac iatrogenic objects that can be seen on CXRs and these have been summarised in Table 2. Radiologists need to recognise them, be aware of their function and look for any related complications.

**TABLE 2:** A list of some of the other non-cardiac iatrogenic materials and medical devices that may be seen on chest radiographs.

Non-cardiac iatrogenic materials and medical devices	Their uses and how to assess them
Haemostatic agents	Use: to control intraoperative bleeding by forming artificial clots and facilitating platelet aggregation.
	As these materials can mimic an abscess or even a tumour on imaging, radiologists need to be aware of their appearance on CXRs (Figure 9).^17,18^
Breast implants	Use: mainly for cosmetic purposes, breast reconstruction post-mastectomy and for correction of congenital malformations.
	Radiologists need to aware of their appearance on CXR (Figure 10). Occasionally the implants may even obscure underlying pathologies in the chest.^19^
Cerebrospinal fluid (CSF) shunts	Use: mainly placed for managing hydrocephalus.
	A basic CSF shunt comprises a proximal catheter, reservoir, valve and a distal catheter. The proximal catheter is placed in one of the lateral ventricles, and it exits through a burr hole, connected to the reservoir in the subcutaneous tissue. The distal catheter can theoretically be placed in any fluid reabsorbing body cavity. Shunts are commonly placed in the peritoneum (ventriculoperitoneal shunt), right atrium (ventriculoatrial shunt) or pleural space (ventriculopleural shunt). Ventriculoperitoneal (VP) shunts are by far the most preferred as they are associated with fewer complications.
	A standard radiographic series (‘shunt series’) includes a frontal and lateral radiograph of the head and neck and frontal radiographs of the chest and abdomen to evaluate the entire shunt (Figure 11a–d). Complications that can be identified on CXR include breaks, disconnections and migrations of the distal catheter, pneumothorax, subcutaneous emphysema and features of pulmonary hypertension.^20^
Vagal nerve stimulator (VNS)	It is the only approved implantable device for long-term management of seizure in patients who are refractory to antiepileptic therapy.
	The device is battery operated and resembles a pacemaker. The device is implanted under the left clavicle. However, unlike a pacemaker or ICD, the lead is positioned in the neck to stimulate the left vagal nerve in the carotid sheath (Figure 12).
	Radiologists need to be aware of these devices so as to not get confused with a pacemaker or ICD.^21^
	Other extra cardiac stimulators that may be seen on CXRs include deep brain stimulation (DBS) devices, bone, diaphragmatic and spinal cord stimulators.^22^
Non-coronary or non-cardiac metallic stents	Uses: for non-coronary vascular applications (e.g. thoracic aorta aneurysm repair [Figure 13], stents for the oesophagus, [Figure 14] trachea–bronchial tree, common bile duct and transjugular intrahepatic portosystemic shunt [TIPS] [Figure 15] for portal hypertension).^23,24^
	As most of the stents are metallic, they are visible on radiographs. It is important that radiologists identify these devices appropriately and evaluate the radiographs for potential complications such as a stent fracture or migration.^25,26^

**FIGURE 13:**
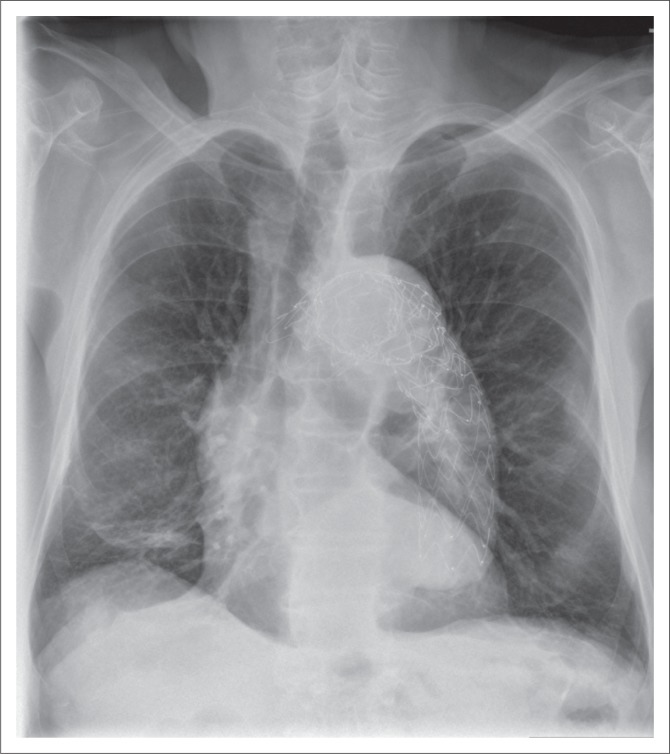
Endovascular stent graft in the thoracic aorta of a 75-year-old man, placed after aneurysm repair.

**FIGURE 14:**
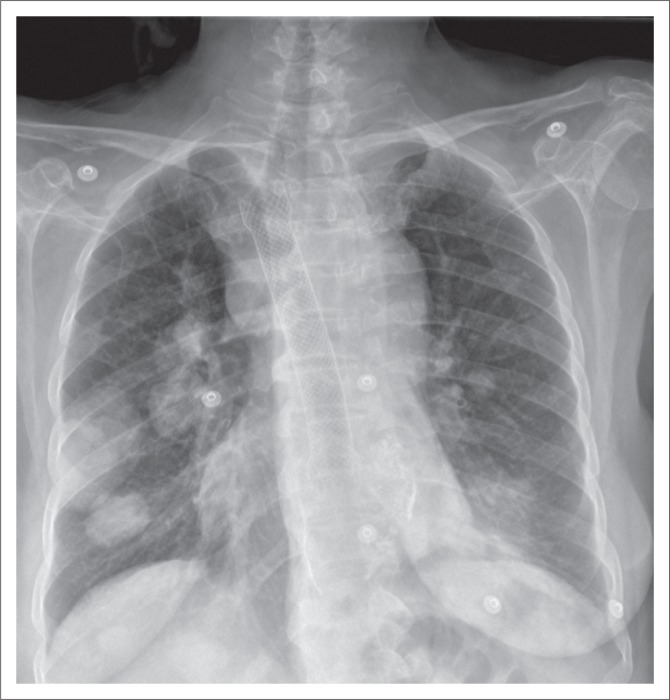
Oesophageal stent inserted in a 68-year-old female with dysphagia secondary to oesophageal carcinoma.

**FIGURE 15:**
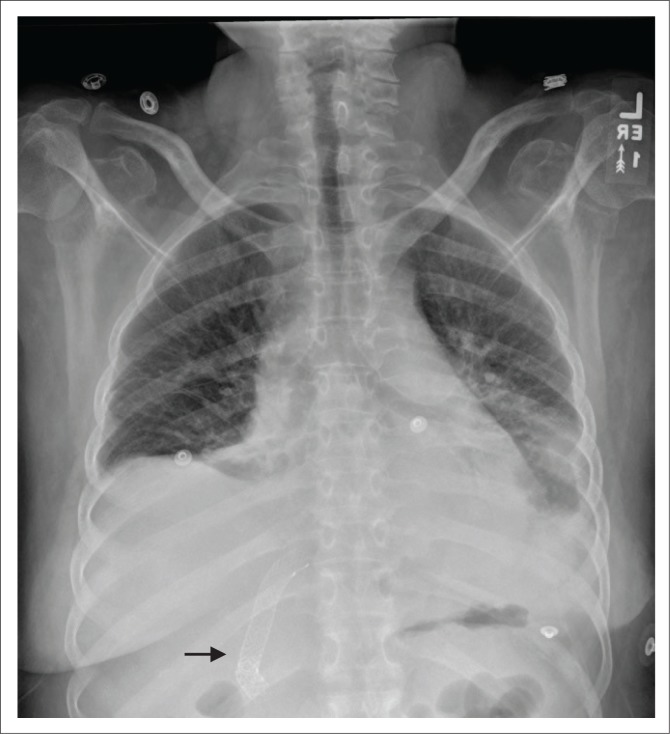
Transjugular intrahepatic portosystemic shunt stent in a 62-year-old female patient with portal hypertension.

## Conclusion

A variety of tubes, lines and medical devices may be seen on CXRs of patients admitted in a hospital. A thorough evaluation of these CXRs is important. Radiologists need to recognise these medical materials, assess them for accurate placement and look for abnormal radiographic presentations, which will enable them to inform the relevant physician or surgeon in a timely manner and help avoid potential consequences.
