# Erratum: Chest radiographs – Lines, tubes, non-cardiac medical devices and materials

**DOI:** 10.4102/sajr.v23i1.1808

**Published:** 2019-12-05

**Authors:** Rishi P. Mathew, Timothy Alexander, Vimal Patel, Gavin Low

**Affiliations:** 1 Department of Radiology and Diagnostic Imaging, Faculty of Medicine and Dentistry, University of Alberta, Edmonton, Canada

In the initial version of this article published earlier, the article title was incorrect. The title is hereby updated as ‘Chest radiographs – Lines, tubes, non-cardiac medical devices and materials’.

This correction does not alter the study’s findings of significance or the overall interpretation of the study results. The publisher apologises for any inconvenience caused.

